# Markers for the detection of Lewy body disease versus Alzheimer’s disease in mild cognitive impairment: a systematic review and meta-analysis

**DOI:** 10.1007/s40520-024-02704-y

**Published:** 2024-03-07

**Authors:** Marianna Ilarj Burgio, Nicola Veronese, Davide Sarà, Carlo Saccaro, Roberta Masnata, Giusy Vassallo, Angela Catania, Giuseppina Catanese, Christoph Mueller, Lee Smith, Ligia Juliana Dominguez, Laura Vernuccio, Mario Barbagallo

**Affiliations:** 1 Department of Health Promotion, Mother Child Care, Internal Medicine and Medical Specialties, University of Palermo, 90127 Palermo, Italy; 2 Geriatric Unit, Azienda Ospedaliera Universitaria Policlinico Paolo Giaccone, Palermo, Italy; 3 International School of Advanced Studies, University of Camerino, Camerino, Italy; 4 South London and Maudsley National Health Service Foundation Trust, London, UK; 5 Institute of Psychiatry Psychology and Neuroscience, Kings College London, London, UK; 6 Centre for Health Performance and Wellbeing, Anglia Ruskin University, Cambridge, CB1 1PT UK

**Keywords:** Lewy body dementia, Alzheimer, Dementia, Mild cognitive decline, Meta-analysis

## Abstract

**Background:**

Mild cognitive impairment (MCI) may evolve into dementia. Early recognition of possible evolution to Alzheimer's disease (AD) and dementia with Lewy Bodies (DLB) is of importance, but actual diagnostic criteria have some limitations. In this systematic review and meta-analysis, we aimed to find the most accurate markers that can discriminate patients with DLB versus AD, in MCI stage.

**Methods:**

We searched several databases up to 17 August 2023 including studies comparing markers that may distinguish DLB-MCI from AD-MCI. We reported data regarding sensitivity, specificity, and the area under the curves (AUCs) with their 95% confidence intervals (CIs).

**Results:**

Among 2219 articles initially screened, eight case–control studies and one cohort study were included for a total of 832 outpatients with MCI. The accuracy of cerebrospinal fluid (CSF) markers was the highest among the markers considered (AUC > 0.90 for the CSF markers), with the AUC of CSF Aβ42/Aβ40 of 0.94. The accuracy for clinical symptom scales was very good (AUC = 0.93), as evaluated in three studies. Although limited to one study, the accuracy of FDG-PET (cingulate island sign ratio) was very good (AUC = 0.95) in discriminating DLB from AD in MCI, while the accuracy of SPECT markers and EEG frequencies was variable.

**Conclusions:**

Few studies have assessed the accuracy of biomarkers and clinical tools to distinguish DLB from AD at the MCI stage. While results are promising for CSF markers, FDG-PET and clinical symptoms scales, more studies, particularly with a prospective design, are needed to evaluate their accuracy and clinical usefulness.

**Clinical trial registration**: Prospero (CRD42023422600).

**Supplementary Information:**

The online version contains supplementary material available at 10.1007/s40520-024-02704-y.

## Introduction

Most forms of dementia are progressive and non-reversible, so the detection of the early stages, such as mild cognitive impairment (MCI), is important. MCI, may represent a target for pharmacologic and non-pharmacologic approaches for slowing the transition to dementia [1]. While Alzheimer’s disease (AD) is the most common form of dementia worldwide, dementia with Lewy bodies (DLB) likely represents up to 20–30% of patients living with dementia [2]. Diagnosis rates are, however, substantially lower in routine clinical services, often less than 5%, meaning that a considerable proportion of DLB diagnoses are missed [3]. A large majority of DLB patients are not often diagnosed and the ascertainment arrives during autopsy [4]. A correct diagnosis is, however, important as DLB has a worse prognosis than other forms of dementia [5] and may permit to give appropriate medications and avoid other solutions (e.g., haloperidol) that can further impair motor aspects [6]. Recently research criteria for MCI in DLB (MCI-LB) have been established, which is an important step to distinguishing DLB from AD already at the MCI stage, and potentially develop tailored interventions. Those research criteria for MCI-LB include a number of features which could distinguish DLB from AD at the MCI stage, as core features of DLB (fluctuating cognition, recurrent visual hallucinations, REM sleep behavior disorder (RBD), at least one Parkinsonian motor sign) and/or proposed biomarkers (reduced dopamine transporter uptake in basal ganglia demonstrated by SPECT or PET, polysomnographic confirmation of REM sleep without atonia, reduced meta-iodobenzylguanidine (MIBG) uptake on myocardial scintigraphy [5]. However, to have markers that may improve the accuracy of DLB diagnosis in MCI stage could be of potential interest since people with DLB usually have different needs from those affected by AD [7]. Given this background, with this systematic review and meta-analysis, we aimed to determine what we already know about the accuracy of biomarkers and clinical scales to discriminate between DLB-MCI and AD-MCI.

## Materials and methods

This systematic review adhered to the PRISMA statement [8, 9] following a protocol available in PROSPERO (CRD42023422600).

### Data sources and searches

Four investigators (MB, DS, AC, GV) in couples, independently, conducted a literature search using PubMed/MEDLINE, Embase, and Web of Science from database inception until 17TH August 2023, including cohort and case–control studies investigating and comparing all the tests and exams that allow to distinguish DLB-MCI) from AD-MCI.

The search terms used in PubMed included combinations of the following keywords: “(mild cognitive impairment OR MCI OR nMCI OR aMCI OR mMCI) AND (Lewy Body Disease OR Lewy Body Dementia OR LBD) AND (Alzheimer Disease OR Alzheimer Dementia OR Alzheimer-Type Dementia OR Alzheimer Type Dementia OR Alzheimer Syndrome OR AD) AND (sensitivit* OR specificit* OR “reproducibility of results” OR predict* OR identif* OR discriminat* OR distinguish* OR differenti* OR diagnos* OR ROC OR receiver operat* OR Area under curve OR AUC OR sROC OR receiving operator curve OR accura*).

### Study selection

Following the PICOS (Population, Intervention, Comparison, Outcomes, Study) criteria, we considered eligible studies that included participants with DLB-MCI detected according to standardized criteria (e.g., Petersen and revised Petersen criteria, McKeith criteria, Matthews criteria, or Clinical Dementia Rating = 0.5) (P), using any kind of marker (e.g., demographic, neuropsychological tests, liquor markers, radiological markers as CT/MRI) (I), versus AD-MCI (C). Regarding the outcomes (O), we included estimates of accuracy (defined as area under the curve [AUC], sensitivity and specificity) or calibration (C-index, pseudo R2, Brier score) in discriminating the two types of MCI. Therefore, cohort and case–control studies were considered (S). We also included a conference abstract if sufficient data were available for the meta-analysis. Exclusion criteria are as follows: (I) Duplicate literature studies, (II) Research with non-human samples, (III) Research without meta-analyzable data (e.g., AUCs without 95% confidence intervals [CIs]), (IV) cognitive impairment not detected by standardized criteria (e.g., only low mini-mental state examination [MMSE] values), (V) healthy controls or other types of dementia such as vascular dementia, as controls, (VI) cross-sectional or case report studies. Following the searches as outlined above, after removal of duplicates, four independent reviewers (MB, GV, DS, AC) screened titles and abstracts of all potentially eligible articles. The authors applied the eligibility criteria, considered the full texts, and a final list of included articles was reached through consensus with a senior author (NV), if needed.

### Data extraction

Two independent investigators (CS, RM) were involved in the data extraction process using a standardized Microsoft Excel database. For each article, we extracted data about authors, year of publication, country/continent, study design, setting, follow up in years (only for the cohort studies), age and its standard deviation, criteria for DLB-MCI and for AD-MCI, percentage of females, total number of patients and of DLB-MCI and AD-MCI.

### Outcomes

The primary outcomes were sensitivity, specificity and the AUCs with their 95% confidence intervals (CIs) of different kinds of markers considered. We also planned to consider data regarding calibration in terms of C-index, pseudo R2, or Brier score, but no study reported this information.

### Assessment of study quality

Based on the revised quality assessment of diagnosis, accuracy studies-2 [10] criteria [10, 11], the included articles were evaluated as at high risk (−) or low risk ( +) by four key domains: patient selection, index test, reference standard, and flow and timing. The evaluation was made by two independent investigators (CS, RM) and checked by another (MB), independently.

### Data synthesis and statistical analysis

We used MedCalc Statistical Software 9.3.8.0 to conduct this meta-analysis, having at least three studies for a marker. Markers using less than three studies were reported descriptively. We calculated the standard error and consequently the pooled AUC with their 95% CIs, applying a random-model effect.[12]The accuracy was then classified using the AUC as very poor (AUC between 0.60 and 0.70), poor (0.70–0.80), good (0.80–0.90), and very good (> 0.90) [13]. Heterogeneity across studies was assessed by the *I*^2^, and a significant heterogeneity was determined by a value *I*^2^ ≥ 50% or the correspondent *p*-value < 0.05 [14]. Publication bias was assessed by visually inspecting funnel plots and using the Egger’s bias test [15], considering a *p*-value less than 0.05 as indicative of publication bias.

## Results

### Study selection

**Fig. 1:**
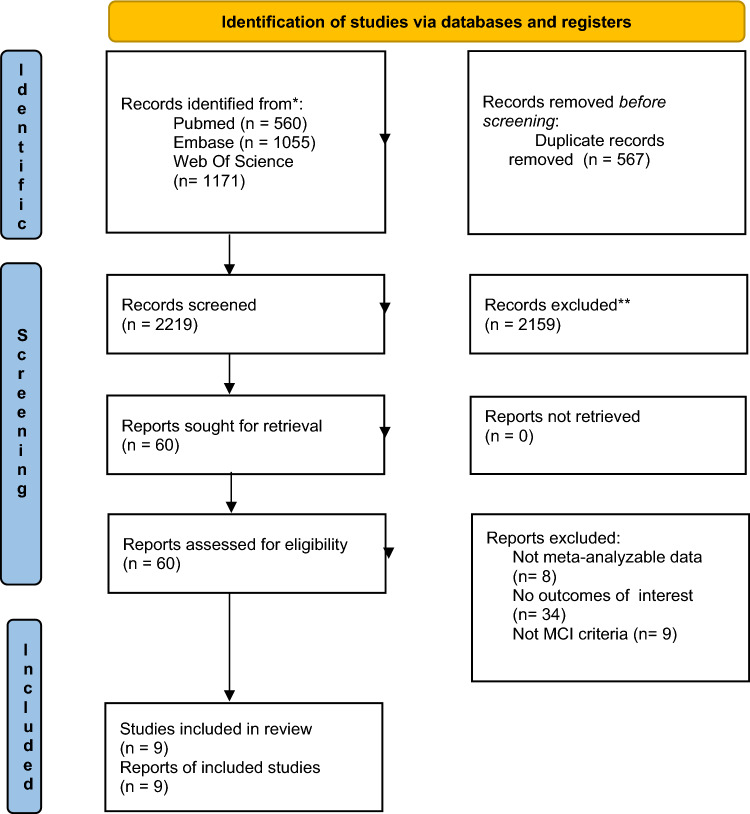
PRISMA 2020 flow diagram

The flow-chart of this systematic review is shown in Fig. 1. Overall, among 2219 papers initially screened, we evaluated 60 full texts. After excluding 51 articles owing to data not meta-analyzable, outcomes of interest were not examined, and MCI criteria were not well specified for the selection of participants (Supplementary Table 1), nine papers were finally included [16–24].

### Descriptive characteristics

Supplementary Table 2, 3 reported the data of the nine works eligible (eight case–control studies and one cohort study) including 832 outpatients. The eight case–control studies included a total of 757 outpatients with diagnosis of MCI according to DSM V and Petersen criteria (*n* = 1), Albert (*n* = 1), CDR (*n* = 2), revised Petersen criteria (*n* = 1) or NIAA-AA criteria (*n* = 3). The mean age at baseline was 72.54 years (SD = 8.74) and 45.22% were female. Of these 757 participants, 398 were diagnosed with AD-MCI according to Albert and Dubois criteria (*n* = 2) McKhann criteria (*n* = 2), Albert criteria (*n* = 1), or Albert, Dubois and McKhann criteria (*n* = 2), NIAA-AA criteria (*n* = 1). DLB-MCI was diagnosed in 359 participants according to DSM V and McKeith criteria (*n* = 2) or McKeith criteria (*n* = 5). Many of the studies were carried out in Europe (*n* = 6), one in America and one in Asia. The only cohort study [25] included a total of 75 outpatients with diagnosis of MCI according to NIAA-AA criteria, with 3 years follow up; the mean age at baseline was 75.37 years (SD = 7.04) and 33.3% were female. Of these 75 participants, 36 were diagnosed AD-MCI according to Albert criteria, 39 participants were diagnosed with DLB-MCI according to McKeith criteria. This study was carried out in the United Kingdom.

### Outcomes of interest

We analyzed sensitivity, specificity, AUCs and their 95% CIs of different markers divided in CSF, neuropsychological, radiologic or EEG markers.

#### CSF markers

As reported in Table 1, almost all the CSF markers showed a very good accuracy in discriminating the two forms of MCI, especially when combined with each other. The combination of *T*-Tau + Ph-Tau + Aβ42/Aβ40 had the highest pooled AUC of three studies[16–18] on a sample size of 179 participants (AUC = 0.96, 95% CI 0.95–0.97, *p*-value < 0.001); the accuracy of Aβ42/Aβ40 was also very good with a pooled AUC of three studies[16–18], on a sample size of 179 participants, of 0.94 (95%CI 0.94–0.95, *p*-value < 0.001). Regarding the combination of *T*-Tau + Ph-Tau + Aβ42, the accuracy was, again, very good: of three studies [16–18] on a sample size of 347 outpatients the AUC was 0.931 (95% CI 0.92–0.93, *p*-value < 0.001). Phospho-tau protein and Tau-protein, even if individually assessed, had a very good accuracy: the estimation of the overall effect of three studies [16–18], on a sample size of 347 outpatients, led respectively to an AUC of 0.93 (95% CI 0.92–0.93, *p*-value < 0.001) and 0.91 (95% CI 0.90–0.91, *p*-value < 0.001). Instead considering Aβ40 and Aβ42 individually, accuracy was poor: the pooled AUC of three studies [16–18] was respectively 0.78 (95% CI 0.77–0.80, *p*-value < 0.001) on a sample size of 179 participants, and 0.78 (95% CI 0.773–0.786, *p*-value < 0.001) on a sample size of 347 outpatients. Even if these findings are limited to only one single study, the accuracy of the combination with *T*-Tau + Ph-Tau + Aβ40/Aβ42 + α-synuclein was very good (44 outpatients, AUC = 0.95, 95% CI 0.83–0.99) [18]. *T*-Tau + Ph-Tau + Aβ42 + α-synuclein had also a very good accuracy on a sample size of 84 outpatients (AUC = 0.95, 95% CI 0.88–0.98)[18]; instead α-synuclein, assessed individually, had a lower but also good accuracy in discriminating the two forms of MCI (84 outpatients, AUC = 0.83, 95% CI 0.73–0.90)[18].

**Table 1:** Main outcomes of case–control studies

Tool	Number of studies	Sample size	AUC	95% CI	*p*-value	I2	*p*-value I2	Egger’s test	*p*-value
T-Tau + Ph-Tau + Aβ42/Aβ40	3	179	0.96	0.95–0.97	< 0.001	59.12	0.08	–2.66	0.004
T-Tau + Phospho-Tau + Aβ42 + α-Synuclein	1	84	0.95	0.88–0.98	< 0.05	NA		NA	
T-Tau + Phospho-Tau + Aβ42/Aβ40 + α-synuclein	1	44	0.95	0.83–0.99	< 0.05	NA		NA	
FDG PET	1	17	0.95	0.75–0.99	0.001	NA		NA	
Aβ42/Aβ40	3	179	0.94	0.94–0.95	< 0.001	0	0.74	–0.71	0.54
T-Tau + Phospho-Tau + Aβ42	3	347	0.93	0.92–0.93	< 0.001	57.7	0.09	1.70	0.45
Ph-tau	3	347	0.93	0.92–0.93	< 0.001	58.34	0.09	0.04	0.98
LBCRS/10-PSS	3	249	0.89	0.83–0.95	< 0.001	97.22	< 0.0001	–11.20	0.08
10 Symptoms scale > 2	2	57	0.91	0.83–0.99	< 0.05	NA		NA	
T-tau	3	347	0.91	0.90–0.91	< 0.001	86.09	0.0008	–0.10	0.98
α-synuclein	1	84	0.83	0.73–0.90	< 0.05	NA		NA	
Aβ40	3	179	0.78	0.77–0.80	< 0.001	73.06	0.02	–4.01	0.08
Aβ42	3	347	0.78	0.77–0.78	< 0.001	97.82	< 0.0001	13.63	0.01
123I-FP-CT-SPECT	1	144	0.76	0.68–0.84	< 0.05	NA		NA	
123I-IMP-SPECT	1	17	0.72	0.4–0.9	0.13	NA		NA	

*Ph-Tau* Phospho-tau, *FDG-PET 18F-*fluorodeoxyglucose positron emission tomography, *LBCRS* Lewy body composite risk score, *10-PSS:* 10-Point symptoms scale, *123I-*FP-CT-SPECT [123I] N-ω-fluoropropyl-2β-carbomethoxy-3β-(4-iodophenyl) nortropane single-photon emission computerized tomography; 123I-IMP-SPECT: 123I-iodoamphetamine single-photon emission computed tomography

#### Clinical scales

The Lewy Body Composite Risk Score Scale (LBCRS) discriminate DLB from all other dementia causes according to the presence or not of suggestive symptoms for at least 6 months or occurring at least three times over the past 6 months[21]. The ten point symptoms scale evaluates the presence or not of symptoms with a prevalence of > 50% of DLB and in < 20% of AD such as fluctuating concentration/attention, episodes of confusion, slack facial expression, drooling, weak voice, hallucinations, involuntary movements, acting out dreams, crying out during sleep, misjudging objects [20, 24]. Regarding the LBCRS and 10-point symptoms scale, using a cut-off > 3, we observed on a sample size a good sensitivity (71.73%) and higher specificity (91.73%). Accuracy was good: the estimation of the overall effect of three studies, on a sample size of 249 outpatients AUC was 0.89 (95% CI 0.83–0.95, *p*-value < 0.001) [20, 21, 24]. Data regarding a 10-point symptoms scale with a cut-off of 1/10 and 2/10 are fully reported in Supplementary Tab. 4.

#### Radiologic markers and EEG markers

The FDG-PET derived CIS ratio had very good accuracy for differentiating the two forms of MCI: AUC was 0.95 (95% CI 0.75–0.99, *p*-value = 0.0018), the sensitivity and the specificity were higher, respectively 77.78 and 100%. [19] On the contrary, ^123^I-iodoamphetamine SPECT-derived CIS ratio was not accurate for differentiating between AD-MCI and DBL-MCI, as shown by AUC 0.72 (95% CI 0.4–0.9, *p*-value = 0.13); sensitivity was 77.78% and specificity was 75% [19]. Also the dopaminergic imaging with ^123^I-FP-CIT SPECT was less useful in identifying DLB-MCI from AD-MCI, with AUC 0.76 (95% CI 0.68–0.84, *p*-value < 0.05); sensitivity was moderate (66%), but specificity was high (88%)[22]. (Table 2).

**Table 2:** Main outcomes of the cohort study

Tool	Number of studies	Sample size	AUC	95% CI	*p*-value
EEG frequency bands: beta power	1	75	0.71	0.59–0.83	0.001
EEG frequency bands: DF, all electrodes	1	75	0.70	0.58–0.82	< 0.001
EEG frequency bands: DF, occipital electrodes	1	75	0.69	0.57–0.81	0.03
EEG frequency bands: pre-alpha power	1	75	0.68	0.56–0.81	< 0.001
EEG frequency bands: alpha power	1	75	0.66	0.53–0.78	0.005
EEG frequency bands: theta/alpha ratio	1	75	0.64	0.51–0.77	< 0.001
EEG frequency bands: theta power	1	75	0.60	0.47–0.73	0.01
EEG frequency bands: delta power	1	75	0.54	0.41–0.67	0.47

*DF* Dominant frequency

In the only cohort study available [23], evaluating the different frequency bands, the results show that the quantitative EEG had a poor accuracy in discriminating the two forms of MCI over three years of follow-up. Sensitivity was high for almost all the frequency bands and the delta power (100%) and alpha power (97%) were greater; however, the specificity was generally much lower, especially for delta (54%) and theta (60%) bands. The greater accuracy was for beta bands with AUC 0.71 (95% CI 0.59–0.83) and dominant frequency with AUC 0.70 (95% CI 0.58–0.82).

### Quality of the studies

The quality of the included studies, as assessed by the QUADAS-2, is reported on Supplementary Table 6. Overall, four studies are at low risk of bias, the other three unclear. The most common source of bias were the index test domain and flow and timing domain, due to the fact that not all patients received the same reference standard and not all patients enrolled were included in the analysis.

## Discussion

In this systematic review with an exploratory meta-analysis including nine studies (eight case–control and one cohort) and a total of 832 older participants, we found that CSF markers are probably the most accurate in discriminating DLB-MCI versus AD-MCI. Other markers and biomarkers considered, such as radiological, EEG and clinical ones, seem to be less accurate. To the best of our knowledge, this is the first systematic review and meta-analysis to explore the usefulness of markers for discriminating between these two forms of MCI. We believe to have reliable biomarkers for discriminating DLB from AD in MCI stage could be of importance in daily clinical practice for several reasons. First, the effect of some medications, such as typical antipsychotics, can lead to a faster clinical worsening and a higher mortality risk in patients with DLB compared to AD [26]. Second, patients with DLB, also in early forms, have a higher risk of some autonomic impairments such as orthostatic hypotension or syncope [27]. Moreover, DLB is usually associated with a worse clinical outcome compared to AD [5] and DLB has specific medical and non-medical needs [28]. Finally, patients with DLB may have more insight into their cognitive deficit compared to AD [29], so, because of the early onset of destructive symptoms (visual hallucinations, fluctuating cognitive function, and REM sleep behavior disorder), they have a decreased Quality Of Life (QoL) compared to patients with AD [29], and the occurrence of depression and nonaccidental self-injury is significantly higher in DLB than in AD [30, 31]. DLB-MCI is a relatively new entity in the topic of dementia. Briefly, in addition to the criteria of MCI, one or more of the core features of DLB are required for a diagnosis of DLB-MCI such as cognitive fluctuations, visual hallucinations, REM sleep behavior disorder, and/or slow or stiff movements. [32] The consensus firstly indicating the importance of DLB-MCI suggests that the use of some biomarkers could be useful for differentiating this entity from AD-MCI, such as dopamine transporter (DAT) imaging, polysomnogram to confirm REM sleep behavior disorder, and a cardiac scan to assess nerve function called MIBG scintigraphy, even if these tests may not have a sufficient sensitivity for detecting the MCI stage of DLB. [32] Therefore, the same authors indicate the importance of more sophisticated tests or biomarkers such as those present in CSF. [32] Our systematic review of case–control studies showed that the combination of several CSF biomarkers such as Aβ42/Aβ40 have an excellent accuracy in discriminating DLB-MCI versus AD-MCI, having an AUC > 0.95. Traditionally, it was reported that Aβ42 levels in CSF are decreased in DLB without significant modifications of other biomarkers usually altered in AD.[17] Regarding Aβ42/Aβ40, it should be acknowledged that CSF Aβ40 levels are usually lower in DLB compared to AD, even if this finding seems to be only in patients with a clinical form of dementia and not MCI.[33] CSF Aβ40 levels, represent the level of amyloid burden in patients affected by dementia[34] similarly to CSF Aβ42 levels that seem to strongly correlate with amyloid plaques and to cognitive severity and consequently evident only in more advance forms of DLB [35]. Unfortunately, we were not able to verify if the alterations of these biomarkers at the baseline can predict any difference in the risk of DLB or AD overtime, since cohort studies are scarce.

Moreover our study evaluated diagnostic accuracy of clinical scale, such as Lewy Body Composite Risk Score and 10-point symptoms scale, for discriminating DLB-MCI instead of AD-MCI [24], 20, 21. Briefly, among ten common symptoms of DLB (i.e., fluctuating concentration/attention, episodes of confusion, slack facial expression, drooling, weak voice, seeing things not present, involuntary movements, acting out dreams, crying out during sleep, and misjudging objects) the presence of three or more have an accuracy of 0.93(sensitivity 71.73% and specificity 91.73%) in predicting the onset of DLB-MCI instead of AD-MCI [20, 21, 24]. However, a limitation of these data is given by the possible presence of heterogeneity (*I*^2^ = 97.22%, *p* < 0.0001). Overall, this study suggests that also in earlier forms differences in clinical aspects are of importance for differentiating DLB-MCI from AD-MCI, so these scales could be a promising markers. Finally, another interesting biomarker could be the presence of the cingulate island sign on 18F-fluorodeoxyglucose positron emission tomography (FDG PET) that seems to have a good accuracy in discriminating DLB-MCI from AD-MCI, even if the use of this biomarker is limited to only one small study of 17 subjects. [19] The only cohort study that we found in our systematic review investigated the use of electroencephalography [35] markers for the discrimination of DLB-MCI instead of AD-MCI, over a median of 3 years of follow-up [25]. This study found that early EEG slowing is a specific feature of DLB-MCI compared to AD-MCI. However, these markers have a good specificity (for alpha waves of 97%), but a very limited sensitivity and accuracy [25].

The findings of our systematic review must be interpreted within its limitations. First, we found only one cohort study and a few case–control studies with limited sample sizes. However, since the biomarkers investigated in each of these studies were expensive or invasive, this limitation could be partially justified. Second, no study tried to directly compare the accuracy of biomarkers having different nature as we did in this systematic review. Finally, the risk of bias was relatively high in all studies included.

In conclusion, CSF markers, particularly Aβ42/Aβ40 seem the most accurate to discriminate DLB-MCI from AD-MCI, although these findings are limited to a few studies. Other biomarkers, such as imaging or EEG seem to be less accurate. Clinical scales appear to have promising accuracy and could be a cost-effective alternative, but more prospective studies are needed to indicate the most efficacious biomarkers and symptom scales for differentiating DLB from AD, also in MCI stage.

### Supplementary Information

Below is the link to the electronic supplementary material.

Supplementary file1 (DOCX 546 KB)

## Data Availability

The databases are available upon reasonable request to the corresponding author.
