# Supplementary material for: Markers for the detection of Lewy body disease versus Alzheimer’s disease in mild cognitive impairment: a systematic review and meta-analysis
Source: Aging Clin Exp Res. 2024 Mar 7;36(1):60. doi: 10.1007/s40520-024-02704-y (PMC10920203; doi:10.1007/s40520-024-02704-y)

**Supplementary Material**

**Supplementary Tab 1. Excluded studies and reasons of exclusion.**

| Not meta-analyzable data | No outcomes of interest | Not MCI Criteria |
| --- | --- | --- |
| 1. *Babilonia, 2018* 2. *Baik, 2022* 3. *Durcan, 2023* 4. *Garçia, 2019* 5. *Kantarci, 2021* 6. *Leko, 2016* 7. *Mc Ardle, 2020* 8. *Mutlay, 2023* | 1. *Barba, 2023* 2. *Bonanni, 2015* 3. *Bousiges, 2015* 4. *Bussè, 2018* 5. *Blanc, 2015* 6. *Blanc, J. Colloby et al. 2016* 7. *Blanc, Roquert et al., 2016* 8. *Cagnin, Bussè, Gardini et al., 2015* 9. *Ciafone, 2022* 10. *Donaghy, 2018* 11. *Donaghy, 2020* 12. *Donaghy, 2022* 13. *Ferman, 2013* 14. *Galvin, 2021* 15. *Hamilton, 2020* 16. *Kantarci, 2016* 17. *Kondo, 2016* 18. *Massa, 2022* 19. *Nedelska, Boeve et al., 2019* 20. *Nedelska, Miyagawa et al., 2019* 21. *O'Caoimh, 2019* 22. *Padovani, 2021* 23. *Payne, 2022* 24. *Perani, 2014* 25. *Quadalti, 2023* 26. *Querry, 2023* 27. *Roberts, Durcan et al., 2018* 28. *Rossi, 2021* 29. *Silva-Rodríguez, 2023* 30. *ShuaiLiu, 2022* 31. *Thomas, 2018* 32. *Thomas, 2021* 33. *Thomas, 2022* 34. *Van De Beek, 2020* | 1. *Cagnin, Bussè et al., 2015* 2. *Ge, 2023* 3. *Hamilton, 2022* 4. *Jicha, 2010* 5. *Kim, 2018* 6. *Mellengaard 2023* 7. *Needelska, 2018* 8. *Olichney, 2005* 9. *Reesink, 2010* |

**Supplementary Tab. 2 Case-control studies: characteristics of included studies**

| **Case Control Studies** |  |  |  |  |  |  |  |  |  |  |  |
| --- | --- | --- | --- | --- | --- | --- | --- | --- | --- | --- | --- |
| **Author, year** | **Country** | **Setting** | **Age at baseline** | **SD [9]** | **% of females** | **Criteria for MCI** | **Criteria for MCI-AD** | **Criteria for MCI-DLB** | **Total MCI (sample size)** | **N MCI-AD** | **N MCI-DLB** |
| **Bousiges (2016)** | France | Outpatients | 67.59 | 8.3 | 44.6 | Petersen, DSM 5 | Albert, Dubois, McKhann | McKeith, DSM 5 | 74 | 39 | 35 |
| **Bousiges (2017)** | France | Outpatients | 69.33 | 8.23 | 50.8 | Petersen | Albert, Dubois | McKeith, DSM 5 | 189 | 132 | 57 |
| **Bousiges (2020)** | France | Outpatients | 68.12 | 8.9 | 50 | CDR | Albert, Dubois, McKhann | McKeith | 84 | 33 | 51 |
| **Chiba (2018)** | Japan | Outpatients | 73.18 | 5.68 | 53 | NIA-AA criteria | McKhann | McKeith | 17 | 8 | 9 |
| **Donaghy (2017)** | United Kingdom | Outpatients | 76.47 | 7.29 | 38.96 | Albert | McKhann | McKeith | 57 | 21 | 36 |
| **Donaghy (2023)** | United Kingdom | Outpatients | 75.7 | 7.54 | 39.67 | NIA-AA criteria | NIA-AA criteria | McKeith | 121 | 51 | 70 |
| **Galvin (2015)** | USA | Outpatients | 76.39 | 8.89 | 53.52 | CDR | Albert,  Dubois | McKeith | 71 | 57 | 14 |
| **Roberts (2021)** | United Kingdom | Outpatients | 75.71 | 7.25 | 31.25 | NIA-AA criteria | Albert | McKeith | 144 | 57 | 87 |
| **Total** | **Europe (n=6); Asia (n=1)**  **USA (n=1)** | **Outpatients (n=8)** | **72.54** | **8.74** | **45.22** | **Albert (n=1); Petersen (n=1); Petersen,DSM V (n=1); CDR (n=2); NIA-AA (n=3)** | **Albert, Dubois (n=2); McKhann (n=2); Albert, Dubois, Mckhann (n=2); Albert (n=1); NIA-AA criteria (n=1)** | **McKeith, DSM 5 (n= 2) ; McKeith (n=6)** | **757** | **398** | **359** |

**Supplementary Tab 3 Cohort study: characteristics of included study**

| **Cohort Studies** |  |  |  |  |  |  |  |  |  |  |  |  |
| --- | --- | --- | --- | --- | --- | --- | --- | --- | --- | --- | --- | --- |
| **Author, year** | **Country** | **Setting** | **Follow up (years)** | **Age at baseline** | **SD [9]** | **% of females** | **Criteria for MCI** | **Criteria for MCI-AD** | **Criteria for MCI-DLB** | **Total MCI (sample size)** | **N MCI-AD** | **N MCI-DLB** |
| **Schumacher (2020)** | United Kingdom | Outpatients | Three years | 75.37 | 7.04 | 33.33 | NIA-AA criteria | Albert | McKeith | 75 | 36 | 39 |
| **Total** | **Europe (n=1)** | **Outpatients (n=1)** | **Three years (1)** | **75.37** | **7.04** | **33.33** | **NIA-AA (n=1)** | **Albert (n=1)** | **McKeith (n=1)** | **75** | **36** | **39** |

**Supplementary Tab. 4 Case-Control studies: data extraction**

| **Tool used** | **Cut-off** | **Sensitivity%** | **Specificity%** | **AUC** | **95% CI low** | **95% CI high** | **Sample size** | **SE** |
| --- | --- | --- | --- | --- | --- | --- | --- | --- |
| ***T-tau*** |  |  |  |  |  |  |  |  |
| *Bousiges (2016)* | >443 | 76.9 | 100 | 0.93 | 0.84 | 0.98 | 74 | 0.008 |
| *Bousiges (2017)* | >443 | 73.5 | 94.7 | 0.91 | 0.86 | 0.94 | 189 | 0.002 |
| *Bousiges (2020)* | > 371 | 81.8 | 88.2 | 0.89 | 0.81 | 0.95 | 84 | 0.007 |
| **Ph-tau** |  |  |  |  |  |  |  |  |
| *Bousiges (2016)* | >62 | 92.3 | 94.3 | 0.94 | 0.86 | 0.98 | 74 | 0.006 |
| *Bousiges (2017)* | >62 | 88.6 | 91.2 | 0.93 | 0.89 | 0.97 | 189 | 0.002 |
| *Bousiges (2020)* | > 60 | 87.9 | 92.2 | 0.92 | 0.84 | 0.97 | 84 | 0.007 |
| **Aβ40** |  |  |  |  |  |  |  |  |
| *Bousiges (2016)* | >9546 | 77.8 | 79.2 | 0.76 | 0.6 | 0.88 | 42 | 0.021 |
| *Bousiges (2017)* | >9546 | 83.3 | 75.8 | 0.8 | 0.71 | 0.88 | 93 | 0.008 |
| *Bousiges (2020)* | > 9563 | 75 | 78.6 | 0.75 | 0.6 | 0.87 | 44 | 0.020 |
| **Aβ42/Aβ40** |  |  |  |  |  |  |  |  |
| *Bousiges (2016)* | ≤0.05 | 88.9 | 100 | 0.95 | 0.83 | 0.99 | 42 | 0.012 |
| *Bousiges (2017)* | ≤0.05 | 78.3 | 100 | 0.95 | 0.88 | 0.98 | 93 | 0.005 |
| *Bousiges (2020)* | ≤0.05 | 81.3 | 96.4 | 0.94 | 0.82 | 0.99 | 44 | 0.012 |
| **T-Tau + Ph-Tau + Aβ42/Aβ40** |  |  |  |  |  |  |  |  |
| *Bousiges (2016)* | > 0,5 | 94.4 | 100 | 0.95 | 0.83 | 0.99 | 42 | 0.012 |
| *Bousiges (2017)* | > 0.5 | 93.3 | 93.3 | 0.97 | 0.91 | 1 | 93 | 0.004 |
| *Bousiges (2020)* | > 0.5 | 87.5 | 100 | 0.95 | 0.84 | 0.99 | 44 | 0.011 |
| **Aβ42** |  |  |  |  |  |  |  |  |
| *Bousiges (2016)* | ≤730 | 84.6 | 71.4 | 0.84 | 0.74 | 0.92 | 74 | 0.010 |
| *Bousiges (2017)* | ≤626 | 68.2 | 79 | 0.76 | 0.69 | 0.82 | 189 | 0.004 |
| *Bousiges (2020)* | ≤ 838 | 81.8 | 76.5 | 0.83 | 0.73 | 0.9 | 84 | 0.009 |
| **T-Tau + Phospho-Tau + Aβ42** |  |  |  |  |  |  |  |  |
| *Bousiges (2016)* | > 0.5 | 89.7 | 97.1 | 0.95 | 0.87 | 0.99 |  |  |
| *Bousiges (2017)* | > 0.6 | 86.4 | 93 | 0.93 | 0.89 | 0.97 | 189 | 0.002 |
| *Bousiges (2020)* | > 0.5 | 81.8 | 96.1 | 0.93 | 0.86 | 0.98 | 84 | 0.006 |
| **α-synuclein** |  |  |  |  |  |  |  |  |
| *Bousiges (2020)* | > 139 | 81.8 | 76.5 | 0.83 | 0.73 | 0.9 | 84 |  |
| **T-Tau + Phospho-Tau + Aβ42/Aβ40+α-synuclein** |  |  |  |  |  |  |  |  |
| *Bousiges (2020)* | > 0.51 | 87.5 | 100 | 0.95 | 0.83 | 0.99 | 44 |  |
| **T-Tau + Phospho-Tau + Aβ42+ α-synuclein** |  |  |  |  |  |  |  |  |
| *Bousiges (2020)* | > 0.48 | 84.9 | 92.2 | 0.95 | 0.88 | 0.98 | 84 |  |
| **LBRCS/ 10-point symptoms scale** |  |  |  |  |  |  |  |  |
| *Galvin (2015)* | >3 | 94.2 | 78.2 | 0.94 | 0.9 | 0.97 | 71 | 0.004 |
| *Donaghy (2017)* | >3 | 62 | 100 | 0.81 | 0.69 | 0.93 | 57 | 0.016 |
| *Donaghy (2023)* | >3 | 59 | 97 | 0.91 | 0.84 | 0.98 | 121 | 0.006 |
| **10-point symptoms scale > 2** |  |  |  |  |  |  |  |  |
| *Donaghy (2017)* | >2 | 83 | 100 | 0,91 | 0.83 | 0.99 | 57 |  |
| *Donaghy (2023)* | >2 | 76 | 90 | 0,91 | 0.84 | 0.98 | 121 |  |
| **10-point symptoms scale >1** |  |  |  |  |  |  |  |  |
| *Donaghy (2017)* | >1 | 90 | 84 | 0.87 | 0.75 | 0.99 | 57 |  |
| *Donaghy (2023)* | >1 | 83 | 83 | 0.91 | 0.84 | 0.98 | 121 |  |
| **123I-IMP-SPECT- derived CIS ratio** |  |  |  |  |  |  |  |  |
| *Chiba (2018)* |  | 77.78 | 75 | 0.72 | 0.4 | 0.9 | 17 |  |
| **123I-FP-CT-.SPECT** |  |  |  |  |  |  |  |  |
| *Roberts (2021)* |  | 66 | 88 | 0.76 | 0.68 | 0.84 | 144 |  |
| **FDG PET-derivet CIS ratio** |  |  |  |  |  |  |  |  |
| *Chibas (2018)* |  | 77.78 | 100 | 0.95 | 0.75 | 0.99 | 17 |  |

**Supplementary Tab. 5 Cohort study: data extraction**

| **Tool used** | **Cut-off** | **Sensitivity%** | **Specificity%** | **AUC** | **95% CI low** | **95% CI high** |
| --- | --- | --- | --- | --- | --- | --- |
| **EEG frequency bands: Delta power** |  |  |  |  |  |  |
| *Schumacher (2020)* | 21.9 | 23 | 89 | 0.54 | 0.41 | 0.67 |
| **EEG frequency bands: Theta power** |  |  |  |  |  |  |
| *Schumacher (2020)* | 10.7 | 33 | 89 | 0.60 | 0.47 | 0.73 |
| **EEG frequency bands: Pre-alpha power** |  |  |  |  |  |  |
| *Schumacher (2020)* | 28.1 | 56 | 83 | 0.68 | 0.56 | 0.81 |
| **EEG frequency bands: Alpha power** |  |  |  |  |  |  |
| *Schumacher (2020)* | 20.5 | 41 | 97 | 0.66 | 0.53 | 0.78 |
| **EEG frequency bands: Beta power** |  |  |  |  |  |  |
| ***Schumacher (2020)*** | 19 | 61 | 81 | 0.71 | 0.59 | 0.83 |
| **EEG frequency bands: Theta/alpha ratio** |  |  |  |  |  |  |
| *Schumacher (2020)* | 0.56 | 49 | 83 | 0.64 | 0.51 | 0.77 |
| **EEG frequency bands: DF, all electrodes** |  |  |  |  |  |  |
| *Schumacher (2020)* | 7.1 | 51 | 86 | 0.70 | 0.58 | 0.82 |
| **EEG frequency bands: DF, occipital electrodes** |  |  |  |  |  |  |
| *Schumacher (2020)* | 7,1 | 51 | 86 | 0.69 | 0.57 | 0.81 |

**Supplementary Tab. 6 Quality assessment of the studies included [10]**


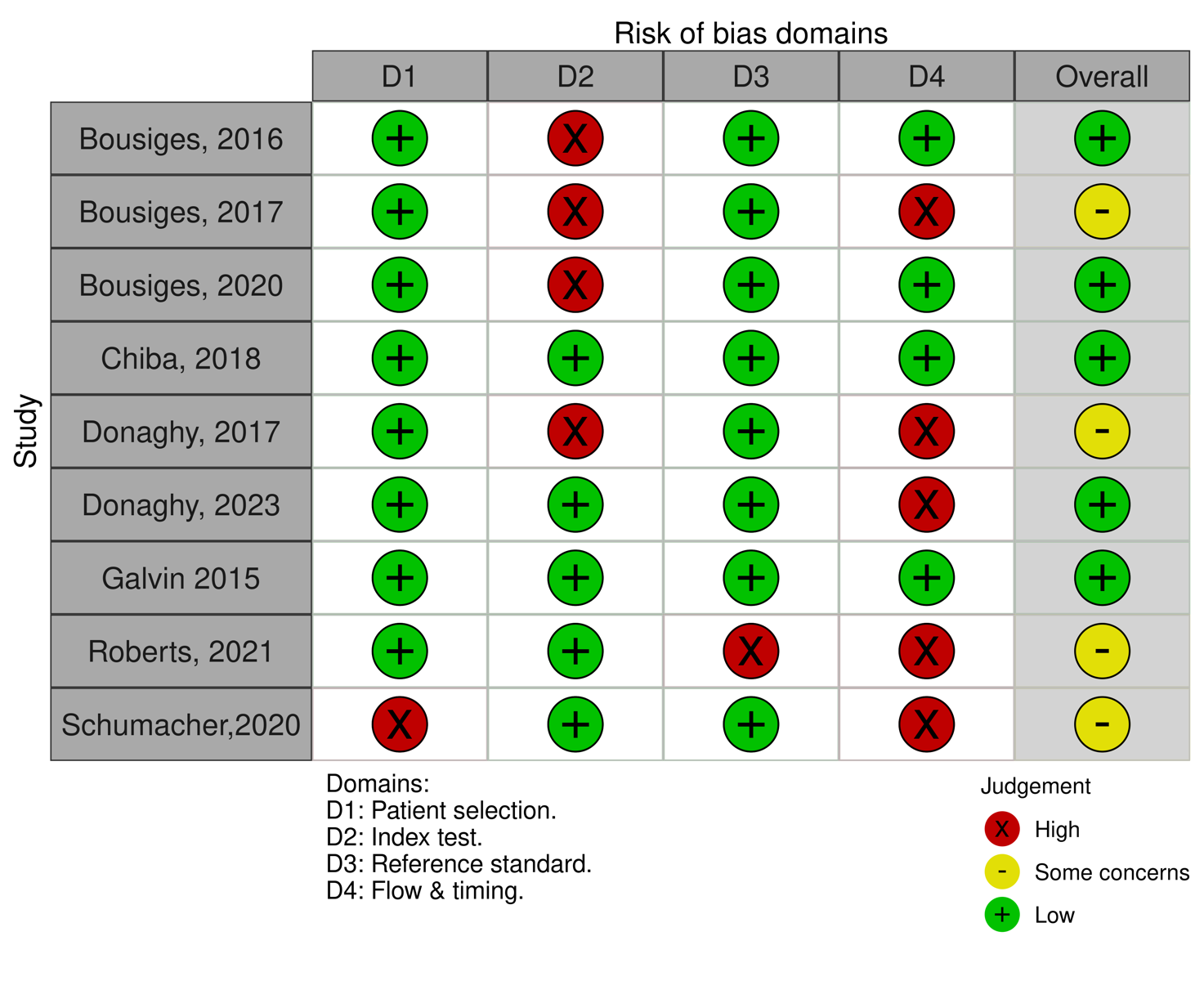


**Supplementary Fig. 1 Forest plot T-Tau**


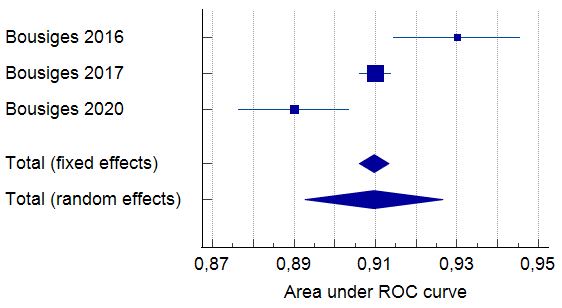


**Supplementary Fig. 2 Forest plot phospho-tau**


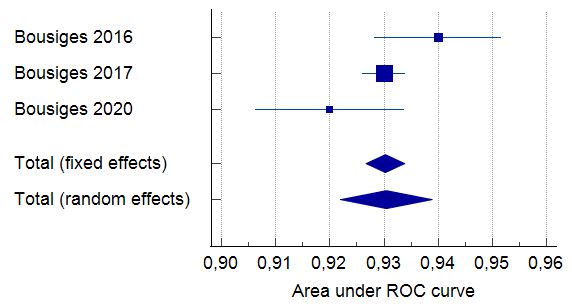


**Supplementary Fig. 3 Forest plot Aβ42**


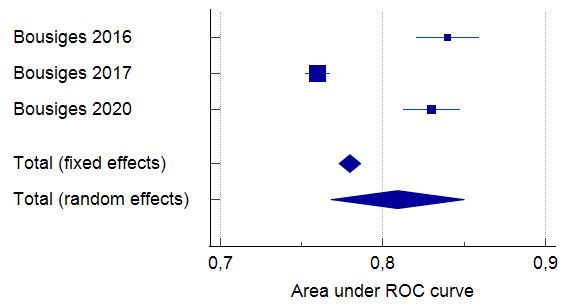


**Supplementary Fig. 4 Forest plot T-tau + phospho-tau+ Aβ42**


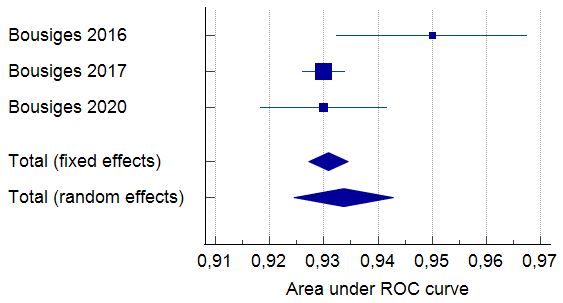


**Supplementary Fig. 5 Forest plot Aβ40**


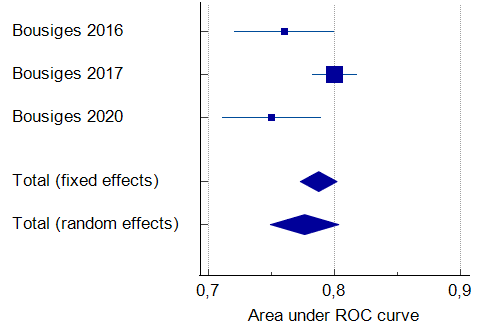


**Supplementary Fig. 6 Forest plot Aβ42/Aβ40**


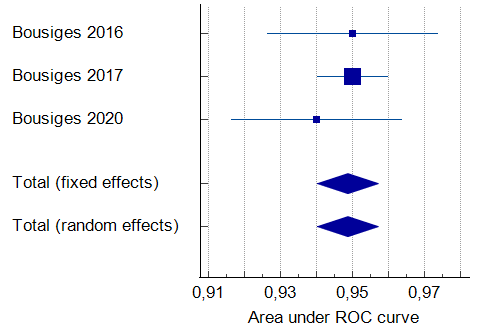


**Supplementary Fig. 7 Forest plot T-tau + phospho-tau+ Aβ42/Aβ40**


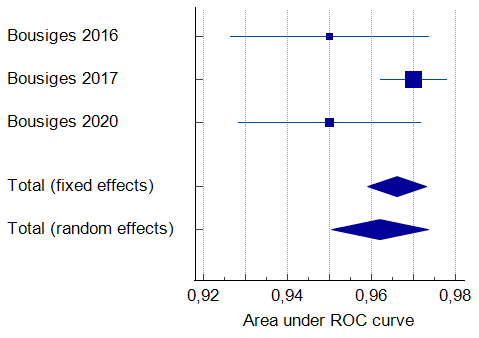


**Supplementary Fig. 8 Forest plot LBCRS/10-PSS**


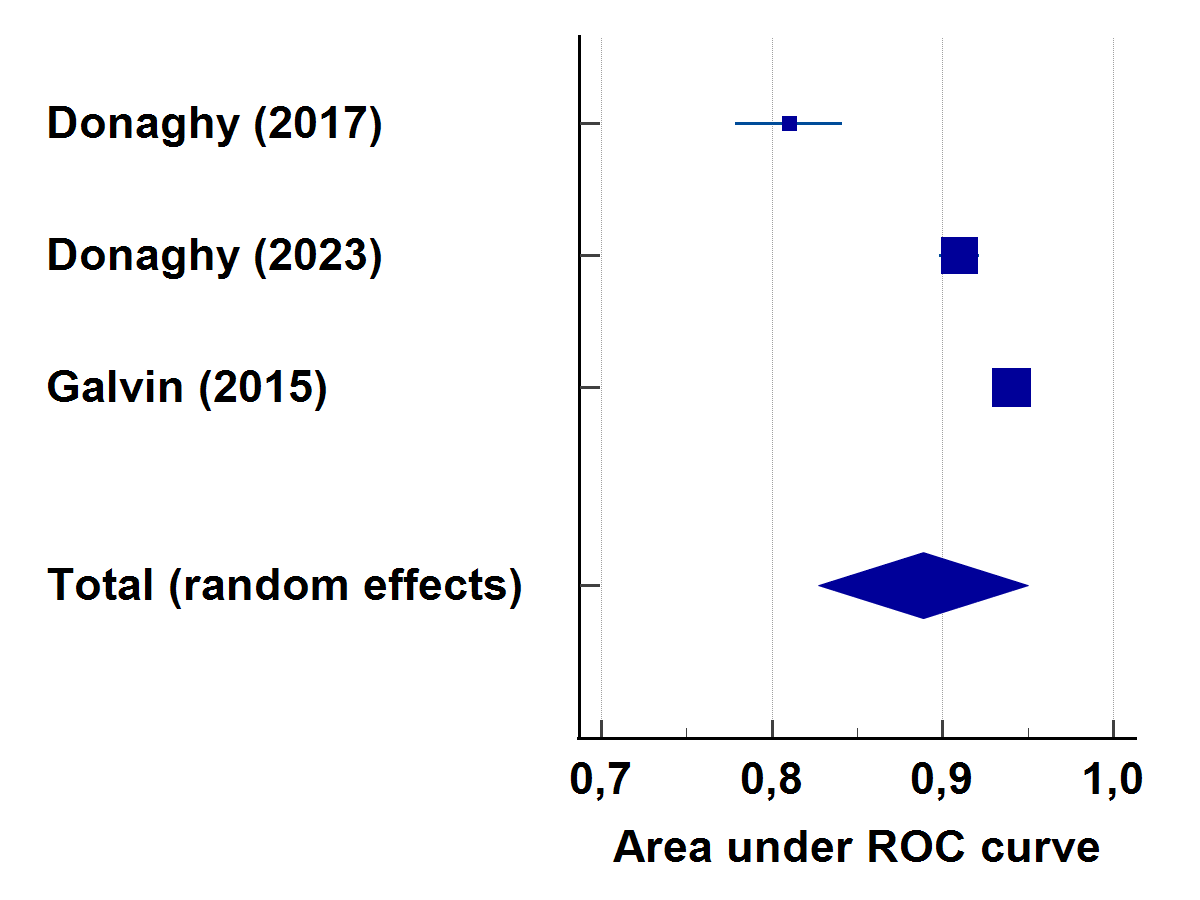

Supplement: Supplementary file 1 — Supplementary file1 (DOCX 546 KB) [file 40520_2024_2704_MOESM1_ESM.docx]
